# Carpal, tarsal, and stifle skin lesion prevalence and potential risk factors in Swiss dairy cows kept in tie stalls: A cross-sectional study

**DOI:** 10.1371/journal.pone.0228808

**Published:** 2020-02-12

**Authors:** Johanna Karin Bernhard, Beatriz Vidondo, Rahel Lisa Achermann, Rahel Rediger, Kerstin Elisabeth Müller, Adrian Steiner

**Affiliations:** 1 Clinic for Ruminants, Vetsuisse-Faculty, University of Bern, Bern, Switzerland; 2 Veterinary Public Health Institute, Vetsuisse-Faculty, University of Bern, Liebefeld, Switzerland; 3 Clinic for Ruminants and Swine, Faculty of Veterinary Medicine, Freie Universität Berlin, Berlin, Germany; Tokat Gaziosmanpasa University, TURKEY

## Abstract

The prevalence of skin lesions at the legs of dairy cows often serves as an indicator for animal welfare and is used as a measurement of adequacy of the present housing conditions. The aim of this study was to assess the prevalence of skin lesions at the carpus, tarsus, and stifle in Swiss dairy cows kept in tie stalls and to describe potential risk factors associated with the different types and severities thereof. Skin lesions and potential risk factors were assessed in 627 cows of 27 tie stall farms in a cross-sectional study. The associations of each outcome and the potential risk factors were assessed by means of logistic regression models using farm as the random factor. One odds ratio was obtained for each biologically relevant risk factor category and the final models were compared between the lesion types and locations. Tarsal lesions were recorded most frequently, with a prevalence of 62.2, 34.4, and 24.0% for moderate to severe hair loss, any severity of ulceration, and moderate to severe swelling, respectively. The prevalence of carpal lesions ranged from 54.4% for hair loss, over 7.7% for ulceration, to 6.1% for swelling, while stifle lesions were recorded less frequently with a prevalence of 18.6, 8.9, 3.4% for hair loss, ulceration, and swelling, respectively. The risk for various skin lesion types and locations significantly increased, when the concrete stall base was covered with a rubber mat and the bedding depth was low. Cows were at the lowest risk to develop skin lesions when they had more than 13 days of outdoor exercise per month. The prevalence of skin lesions in tied Swiss dairy cows is remarkably high and could possibly be reduced by providing the herd more frequent outdoor exercise and a well-cushioned, friction-absorbing and non-abrasive lying surface.

## Introduction

Skin lesions at exposed, periarticular areas of cows' legs are a frequently reported issue in dairy production and affect the welfare [1–3] and productivity [4] of dairy cows. The prevalence of skin lesions in a dairy herd represents a critical area of dairy cow comfort in tie stalls [5]. Although the trend in modern dairy production is moving towards free stall housing, still about 39 to 75% of US and Canadian dairy farms use [6, 7], and about 40% of Swiss dairy cows are kept in tie stalls [8]. However, the reported skin lesion prevalence and the described risk factors from North-American tie stalls [9–13] are difficult to transfer to traditional Swiss tie stalls, as herd size, dairy cow genetics, stall design, and management factors, i.e. access to pasture or outdoor pens, differ largely.

Since the latest investigation in Switzerland [14], tie stall facilities needed to be adapted according to updated national regulations [15], feeding practices got more intensive and milk yield increased. Furthermore, the awareness of the society, politics, and farmers towards animal welfare grew. Labels with stricter requirements with respect to animal welfare were implemented, including the Swiss national program for regular outdoor exercise [16]. Exercise outdoors is known to result in improved animal welfare [17, 18], fewer skin lesions [1, 14, 19, 20], and fewer lameness cases [1]; although, it is often unclear, if lameness is the cause or the effect of a skin lesion [21–23]. Besides lameness [4, 20, 24, 25], body condition score [14], breed [19, 26], lactation stage [12, 27, 28] and number [14, 25], and cow cleanliness [11, 14, 29] are reported to be associated with skin lesions. Likewise, environmental factors can play a major role in lesion development; mainly inadequate tie stall design features [28, 30]. In Swiss tie stalls, the stall base mostly consists of blank concrete which is often covered with a rubber mat. Although, different types of rubber mats are approved for use in Swiss dairy operations [31], solid rubber mats are used primarily. The use of bedding material in tie stall operations is statutory in Switzerland [32], because the presence of bedding may decrease the risk for skin lesions [14, 28], increase cow comfort [33] and raise the cows' preference to lie down in the stall [34].

Although animal welfare, housing and management conditions of Swiss dairy herds have continuously been improved during the past years, skin lesions still being present at the carpal, tarsal and stifle joints are expected to compromise the animal welfare in tied Swiss dairy cows. The assessment of a current prevalence status might increase the farmers' awareness towards such lesions as an animal welfare indicator, while the knowledge about specific risk factors in traditional Swiss tie stalls is crucial to develop customized recommendations for improvement.

To score the type and severity of skin lesions, multiple scoring systems were established in the past. Either the presence of hair loss, ulceration, and swelling was expressed in a single score per joint [35, 36], or the different lesion types were scored separately [2, 29]. The separate scoring of each lesion type allows to individually assess associations between each potential risk factor and the respective lesion type and enables to compare the influence of the risk factors on each of the different outcomes. This might create additional knowledge about the etiology of skin lesions, which is still not entirely clear. Some researchers assume a progression from hair loss over ulceration to swelling [23], while others regard these lesion types at least in parts as individual clinical manifestations [29]; however, inadequate housing in general is described as a main cause of all lesion types [28]. Skin lesions around protruded bony structures are mostly caused by chronic mechanical irritation [23], while hair loss alone is assumed to be an indicator of mild abrasion which is not severely affecting animal welfare [37]. Ulceration and swelling are signs of inflammation, and therefore are considered more severe than hair loss alone [38].

The aim of the present study was to assess the prevalence for carpal, tarsal, and stifle lesions in Swiss dairy cows kept in tie stalls and evaluate the influence of potential risk factors on the development of hair loss, ulceration, and swelling of the skin surrounding these joints.

## Materials and methods

### Ethical approval

This study was conducted as a part of a larger tie stall project of the Vetsuisse-Faculty Bern. Ethical approval was obtained from the Veterinary Office of the Canton Bern (Switzerland; approval no. 29518) for the following cantons: Bern, Aargau, Fribourg, Jura, Vaud, Luzern, Schwyz, Uri, Nidwalden, Obwalden, Neuchâtel, Solothurn.

### Recruitment and farm visits

To recruit participants for the study, two newspaper articles were published in agricultural magazines. The articles aimed to inform Swiss farmers about the concern of lameness and skin lesions in dairy herds and the implementation of the study. Additionally, letters were sent to the members of the Swiss tie stall association, including an information notice about the study and the invitation to participate. Interested farmers contacted the first author by telephone or email and received further information about the project. To be included in the study, farms needed to be localized in one of the cantons the study got ethical approval for, and to keep at least fifteen lactating cows in a tie stall. All farmers participated voluntarily and were selected by convenience.

Between December 2017 and April 2018, each farm was visited once. The current study was conducted as a cross-sectional, observational, analytical study. The farm visit took place at least 4 weeks after the winter housing period started; the order of farm visits was discerned to be prior to the next claw trimming appointment, which was necessary in the course of a larger tie stall project, of which the current study is part.

### On-farm assessment

All on-farm assessments were conducted by one veterinarian (first author) and at least one of two final-year veterinary students (third and fourth author). The skin lesion scoring was consistently performed by the first author. All on-farm assessments were conducted while the cows were standing in their stalls; except for carpal lesions that were scored as the cows were standing in a claw-trimming chute, ensuring safety for the investigator and good light conditions to score the skin lesions. Laminated definition cards and standard operating procedures were used to ensure a high repeatability of scorings and measurements at all time, an example of which can be found in S1 Fig.

All lactating and dry cows that were present in the tie stall the day of the farm visit were examined for hair loss, ulceration, and swelling at both left and right carpus, tarsus, and stifle. The 4-point-scale according to Potterton et al. [29] was used to assess the severity of each tarsal lesion type. The scoring system was applied in the same manner and with the same cut-off values for carpal and stifle lesions. Hair loss and ulceration were scored on the dorsal aspect in carpal joints, and on the lateral aspect in tarsal and stifle joints. The maximum diameter of each lesion was estimated using a hand-held measuring bar and indicated the severity of the lesion (absent (score 0), lesion diameter of approximately <2 cm (score 1), lesion diameter of 2–2.5 cm (score 2), lesion diameter >2.5 cm (score 3)). Visual assessment of swellings was performed from the lateral aspect in carpal joints, and from the caudal aspect in tarsal and stifle joints and scored as absent (score 0), mild (score 1), moderate (score 2), or severe (score 3). If a cow was too dirty to ensure a correct lesion scoring, the area surrounding the respective joint was cleaned prior to lesion assessment.

The type of tying system, stall base and bedding material was visually assessed, as well as the presence of a neck rail and a rail at the rear curb of the lying surface, which is used to keep the bedding material in place.

To assess the environment associated factors bed length, bed width, manger depth, lunge space, and curb height, the first, middle, and last usable stall per stall row were measured (S2 Fig). The mean value per stall row was calculated for each factor and considered for the statistical analysis. For cow or environment associated factors that were more likely to vary individually, fifteen cows per herd and the belonging stalls were randomly selected prior to the farm visit. The cow cleanliness score (adapted from Bouffard et al. [9]), body condition score [39], withers height, bedding depth, bed cleanliness, and stall wetness were measured in these selected cows and stalls. The median value per stall row was calculated for each factor and considered for the statistical analysis.

Bed length was measured along the longitudinal midline of the stall, and defined as the space from the cow-facing border of the manger wall to the end of the lying surface, which was either a rear curb or a rail. Bed width was measured at the level of the manger wall and was defined as the space between two dividing elements. The manger depth was defined as the distance from the upper border of the manger wall to the base of the manger, measured at the longitudinal midline of the stall, representing the distance the cow needed to bend down while feeding. Lunge space was defined as the space without any obstruction from the manger curb forward, measured at the longitudinal midline of the stall. If the non-obstructed space was infinite, e.g. when the manger was designed as a feed alley, lunge space was set to 100 cm; if the access to the manger was prohibited during several hours per day, lunge space was set to 0 cm. The rear curb height was defined as the distance between the lying surface to the next fortified area in the rear of the box. A schematic illustration of the stall measurements is presented in S3 Fig.

The cow cleanliness score (CCS, adapted from Bouffard et al. [9]) was assessed for the following three areas: the lateral aspect of the right flank (area between virtual line between pin and hook bones and middle tarsal joint) and lower hind leg (area between middle tarsal joint and coronary band), and the caudal aspect of the udder (lower 50% of the udder, excluding the teats). The scoring criteria are displayed in Table 1. The sum of scores of the three areas in each cow was considered for the statistical analysis. The body condition score (BCS) was assessed on a 5-point-scale with 0.25 intervals [39]. Withers height was measured at the height of the front limb, from the lying surface to the top line of the cow. The calculated ratio of the withers' height over bed length and withers' height over the product of bed length and bed width was used to investigate associations between the space allowance per animal and the outcome. The bedding depth, bed cleanliness and stall wetness were measured at those two points of each stall, where the legs were suspected to get in contact with the lying surface when the cow was lying down. Both points were located at the longitudinal midline of the stall, point A was measured 50 cm from the manger curb backward, point B was measured 50 cm from the rear of the stall forward. The bedding depth was rated as ≤2 cm or >2 cm [40]. The stall wetness was measured as the investigator kneeled on a folded kitchen paper towel for 3 seconds and evaluated by the size and distribution of wet areas. A 3-point-scale describes a dry, moist, or wet lying surface [41]. The bed cleanliness was recorded on a 4-point-scale, where the lying surface was clean, contaminated with <50% of manure, ≥50% of manure, or entirely contaminated [41].

**Table 1:** Cow cleanliness score (adapted from Bouffard et al. [9]), assessed on the lateral aspect of the right flank and lower hind leg, and the caudal aspect of the udder.

Score	Description
CCS 0	<50% of the area contaminated with fresh splashes of manure, no dried manure
CCS 1	≥50% of the area contaminated with fresh splashes of manure, no dried manure
CCS 2	<50% of the area contaminated with dried manure, area may be contaminated with fresh splashes of manure
CCS 3	≥50% of the area contaminated with dried manure, area may be contaminated with fresh splashes of manure
CCS 4	whole area contaminated with dried manure

CCS: Cow Cleanliness Score

In the course of a larger tie stall project investigating the same study population, 592 of 609 cows underwent a live stall lameness scoring [42] at the day of the farm visit, evaluated by the first author. The cows were observed one by one for unequal weight bearing while stepping from side to side, resting a foot while standing, shifting weight from one foot to the other, and standing at the edge of the rear curb. If a cow showed at least two of the defined behavioral patterns during the observation period of 90 seconds, she was considered to be lame, if not, she was considered to be non-lame. Furthermore, 543 of 609 cows were videotaped while being walked in a straight line by the farmer; the videos were independently scored by three trained observers (first, third and fourth author) according to Flower and Weary [43]. An inter-observer variation of ≤1.0 was accepted, so that a mean locomotion score was calculated for each examined cow. Cows with a mean locomotion score of <2.25 were considered to be non-lame; cows with a mean locomotion score of ≥2.25 were considered to be lame. If the inter-observer variation was >1.0, the three observers scored the videos again, and an acceptable agreement was reached (n = 2).

The following parameters were derived from the national animal database: individual ear tag number, breed, date of birth, date of purchase, parity, days since last calving.

Each farmer was interviewed by the first author at the beginning of the farm visit to collect information about herd and management associated factors. The questionnaire contained single and multiple choice, as well as short answer questions. The farmer was asked how many days per winter month he provided the dairy herd outdoor access. Furthermore, he was asked how often he would feed the cows per day, if he would fulfill the requirements of a certain label with stricter requirements with respect to animal welfare, and if he would clip the hair of the cows hind limbs at the beginning of the winter housing period. This is common practice in Swiss dairy operations to make the cows' fur less adhesive for manure, keep the cows clean, and prepare them for exhibitions.

Lactating and dry cows that were not present in one of the tie stalls of the farm at the day of the farm visit for individual management reasons were excluded from the study. If one of these cows was randomly selected in advance for additional measurements, the next cow on the list that was present in the tie stall was vicariously chosen. Furthermore, cows that were purchased less than 6 weeks before the day of the farm visit were excluded from the statistical analysis.

### Data handling

On-farm assessments were documented on paper, and later entered into a Microsoft Excel (version 2016, Microsoft Corp.) datasheet and audited. The questionnaire data were directly entered into a Microsoft Access (version 2016, Microsoft Corp.) datasheet on a tablet PC, exported to Microsoft Excel (version 2016, Microsoft Corp.) and merged with the main dataset.

### Statistical analysis

Data were analyzed in RStudio 1.1.463 (RStudio Team 2015. RStudio, Inc., Boston) and STATA 15.1 (StataCorp. 2017. College Station, TX: StataCorp LLC).

In each cow, both left and right limbs were scored for the three skin lesion types, but only the more severe score per lesion type and location was forwarded to the analyses. Due to the outcome distribution (Table 2) and the biological relevance, all skin lesion types were dichotomized. Carpal and tarsal skin with absent or mild hair loss (score 0 and 1) was considered non-injured, and with moderate to severe hair loss (score 2 and 3) injured. This classification was also applied for swelling at the carpus and tarsus. Any presence of ulceration (score 1 to 3) was considered injured and only an intact skin surface (score 0) was considered non-injured. Because stifle lesions in general were expected to appear rarely, skin lesions of any type and severity (score 1, 2, and 3) were considered injured, and only the absence of any lesion (score 0) was considered non-injured. The study population's prevalence of hair loss, ulceration, and swelling was calculated at both the cow and the herd level for each assessed location. Confidence intervals (CI_95%_) of the population's prevalence were estimated assuming a binomial distribution [44].

**Table 2:** Distribution of carpal, tarsal, and stifle lesion scores in 609 cows from 27 farms.

	Carpal lesion			Tarsal lesion			Stifle lesion		
	Hair loss n_cows_ (%)	Ulceration n_cows_ (%)	Swelling n_cows_ (%)	Hair loss n_cows_ (%)	Ulceration n_cows_ (%)	Swelling n_cows_ (%)	Hair loss n_cows_ (%)	Ulceration n_cows_ (%)	Swelling n_cows_ (%)
Score 0	209 (34.4)	560 (92.3)	319 (52.6)	151 (24.8)	399 (65.5)	37 (6.1)	496 (81.4)	555 (91.1)	588 (96.5)
Score 1	68 (11.2)	42 (6.9)	251 (41.4)	78 (12.8)	141 (23.2)	426 (70.0)	30 (4.9)	25 (4.1)	15 (2.5)
Score 2	103 (17.0)	5 (0.8)	35 (5.7)	128 (21.0)	58 (9.5)	138 (22.6)	32 (5.3)	24 (4.0)	4 (0.7)
Score 3	227 (37.4)	0 (0.0)	2 (0.3)	252 (41.4)	11 (1.8)	8 (1.3)	51 (8.4)	5 (0.8)	2 (0.3)

n_cows_: absolute number of cows

The data distribution of each potential risk factor was investigated using descriptive statistics, visually checked for normality with histograms and statistically with a Shapiro-Wilk test (p <0.05). Subsequently, the association of each outcome with selected potential risk factors was assessed by means of logistic regression mixed models using farm as the random factor and the cow as the unit of analysis. First, null-models and univariable models were fit and then multivariable models were constructed using biologically meaningful combinations of the significant risk factors. To better address non-linear associations of the outcomes with continuous risk factors, continuous risk factors were transformed into categorical variables. In this way, instead of obtaining a single Odds Ratio (OR) that informs on the increase of risk of the outcome per unit of continuous variable, one OR per category was obtained. Depending on the distribution of the continuous variables, they were grouped into two or four categories defined by their median and their quartiles. If two potential risk factors showed a linear correlation (Pearson's r >|0.7|), only the biologically more relevant factor was included in the analysis. If both were equally relevant, only the risk factor the strongest related to the outcome in the univariable analysis was forwarded to the multivariable model.

The level of significance for the univariable logistic regression models was set to 0.2 in order not to miss any important association. Significantly associated risk factors were then chosen by manual stepwise forward selection in the multivariable logistic regression model. The level of significance for the final models was set to 0.05. After each new variable was added, a likelihood-ratio test was used to determine if the variable added last improved the model fit. The model fit of the multivariable models was evaluated with a Wald-Chi^2^ test. Additionally, every two-level model was compared to its one-level equivalent with a likelihood-ratio test. If multiple models were comparable, the biologically more meaningful model was selected; models that were eliminated in this process are presented in S2 Table and are not further described in this manuscript. For each final model, the variance partition coefficient [45] that informs on the amount of variance existing at the farm level was calculated.

## Results

### Study population

Between December 2017 and April 2018, a total of 27 farms in the cantons Bern (n = 19), Luzern (n = 5), Solothurn (n = 1), Aargau (n = 1), and Uri (n = 1) were visited. Within these herds, 627 cows were assessed for skin lesions; whereof 18.5, 20.7, 30.5, 22.2, and 8.1% of cows were enrolled in December, January, February, March, and April, respectively. After exclusion of newly purchased cows (n = 18), complete records of 607 cows for carpal, and 609 cows for tarsal and stifle lesions were included in the statistical analysis. All represented breeds were combined to three categories according to the estimated milk yield of the breed [46] (Table 3). The study population was consisting of 549 (90.1%) lactating and 60 (9.9%) dry cows. Herd size ranged from 15 to 49 adult cows with a median (interquartile range) herd size of 22 (20–30) cows per herd. Farmers indicated to provide their herd outdoor access at 4 to 24 days per month, with a median (interquartile range) of 13 (13–15) days of outdoor access per month.

**Table 3:** Categorization of represented breeds and distribution among the study population, due to the estimated milk yield of each breed (Keil et al., 2006).

Category (%)	Breed	n_cows_ (%)
Dairy (35.8)	US-Brown Swiss	93 (15.3)
	Holstein Friesian	57 (9.3)
	Jersey	3 (0.5)
	Red Holstein	65 (10.7)
Dual-purpose (21.5)	Original Swiss Brown Cattle	23 (3.8)
	Simmental	108 (17.7)
Crossbreed (42.7)	Swiss Fleckvieh (Holstein x Simmental)	146 (24.0)
	Rotfleckvieh	102 (16.7)
	Dairy crossbreed	12 (2.0)

n_cows_: absolute number of cows

All lactating cows were milked twice daily. 245 (40.2%) cows were fed at least three times daily; 233 (38.3%) cows were tied with a split chain (180°-rotated Y-shaped chain). Due to their variability, other tying systems, such as single chains to the manger curb, side or neck rail, or systems with vertical wooden bars (traditional tying system) were categorized as other. The concrete stall base was covered with a rubber mat in 489 (80.3%) stalls, blank concrete was present in 120 (19.7%) stalls. A bedding depth of >2 cm was recorded in 75 (12.3%) of stalls at point A, and in 189 (31.0%) of stalls at point B. Of the cows assessed for lameness, 75 (12.7%) cows were considered to be lame according to the results of the stall lameness scoring [42], while 80 (14.7%) were considered to be lame according to the locomotion scoring [43].

### Prevalence of carpal, tarsal, and stifle lesions

#### Herd level prevalence of skin lesions

In almost all investigated herds (96.3%; CI_95%_ 81.0–99.9%) at least one cow with moderate to severe hair loss at the carpus was present. In more than half of the herds (55.6%; CI_95%_ 35.3–74.5% and 48.1%; CI_95%_ 28.7–68.1%, respectively) at least one cow with a carpal ulceration or swelling was recorded. All investigated herds (100.0%; CI_95%_ 87.2–100.0%) had at least one cow with at least one moderate or severe hair loss at the tarsus. Ulceration and swelling at the tarsus were recorded in the majority of herds (92.6% for both; CI_95%_ 75.7–99.1%). The proportion of herds that had at least one cow with a stifle lesion was high for hair loss (81.5%; CI_95%_ 61.9–93.7%), moderate for ulceration (63.0%; CI_95%_ 42.4–80.6%), and lowest for swelling (33.3%; CI_95%_ 16.5–54.0%).

#### Cow level prevalence of skin lesions

Detailed information about the distribution of outcomes on the cow level is provided in Table 2. The cow level prevalence of hair loss at the carpus was rather high, as the majority of cows (54.4%; CI_95%_ 50.3–58.4%) was affected. Ulceration at the carpus was recorded less frequently (7.7%; CI_95%_ 5.7–10.2%); the lowest prevalence of carpal lesions was found for swelling (6.1%; CI_95%_ 4.3–8.3%). Moderate to severe hair loss at the tarsus was diagnosed in the majority (62.4%; CI_95%_ 58.4–66.3%) of included cows. One third of cows (34.5%; CI_95%_ 30.7–38.4%) presented with ulcerated skin at the tarsus, and one quarter (24.0%; CI_95%_ 20.6–27.6%) presented with moderate to severe swelling. At the stifle, mild, moderate or severe hair loss was recorded in about one fifth (18.6%; CI_95%_ 15.5–21.9%) of the investigated cows, while ulceration and swelling of any severity were recorded rarely (8.9%; CI_95%_ 6.7–11.4% and 3.4%; CI_95%_ 2.1–5.2%, respectively).

### Potential risk factors associated with carpal, tarsal, and stifle lesions

The potential risk factors curb height and presence of a rear rail showed a linear correlation (correlation matrix can be found in S2 Table), thus only the risk factor the strongest related to the outcome in the univariable analysis was considered for the respective final models.

Detailed information about the final models for carpal lesions are presented in Table 4. For all carpal lesion types, the risk of presenting with a lesion was lowest in December, increased in January, and either leveled off (hair loss), fluctuated (ulceration), or continuously raised (swelling) until April. Frequent exercise outdoors decreased the risk for hair loss and ulceration, and a lunge space of >73 cm decreased the risk for ulceration and swelling at the carpus. A bedding depth of >2 cm significantly decreased the risk for hair loss at the carpus. The variance partition coefficient (VPC) of the final carpal models ranged from 8.4, over 9.8, to 13.0% for the farm level for the outcomes hair loss, ulceration, and swelling, respectively.

**Table 4:** Final multilevel logistic regression model for carpal lesions with cow and herd level risk factors in 27 tie stall farms (n = 607 cows) in Switzerland.

Carpal lesion	Variable	Category	Coefficient	SE	OR	CI_95%_		P-value
**Hair loss**	**Month of visit**	December	Reference	-	-	-	-	-
		January	2.36	0.53	10.61	3.78	29.74	<0.001
		February	2.16	0.51	8.63	3.17	23.46	<0.001
		March	1.92	0.54	6.79	2.37	19.48	<0.001
		April	1.02	0.71	2.77	0.69	11.05	0.150
	**Bedding depth (point A)**	≤2 cm	Reference	-	-	-	-	-
		>2 cm	-0.88	0.39	0.41	0.19	0.89	0.024
	**Outdoor access (days/month)**	<13	Reference	-	-	-	-	-
		13	-0.23	0.43	0.79	0.34	1.86	0.590
		14–15	-1.24	0.50	0.29	0.11	0.77	0.013
		>15	-0.70	0.59	0.49	0.15	1.58	0.234
	Constant		-0.94	0.58				
	Variance herd level		0.36	0.19				
**Ulceration**	**Month of visit**	December	Reference	-	-	-	-	-
		January	2.51	1.21	12.36	1.14	133.61	0.038
		February	1.98	1.17	7.22	0.73	71.41	0.091
		March	2.55	1.15	12.77	1.34	121.45	0.027
		April	1.50	1.34	4.49	0.33	61.66	0.261
	**Free lunge space**	≤73 cm	Reference	-	-	-	-	-
		>73 cm	-2.36	0.56	0.09	0.03	0.29	<0.001
	**Outdoor access (days/month)**	<13	Reference	-	-	-	-	-
		13	-1.59	0.62	0.20	0.06	0.69	0.011
		14–15	-2.42	0.77	0.09	0.02	0.41	0.002
		>15	-3.41	1.29	0.03	0.00	0.42	0.008
	Constant		-2.44	1.25				
	Variance herd level		0.30	0.31				
**Swelling**	**Month of visit**	December	Reference	-	-	-	-	-
		January	1.61	1.32	4.99	0.38	66.04	0.223
		February	2.37	1.16	10.71	1.11	103.26	0.040
		March	2.42	1.18	11.23	1.12	112.75	0.040
		April	2.59	1.27	13.36	1.11	160.91	0.041
	**Free lunge space**	≤73 cm	Reference	-	-	-	-	-
		>73 cm	-1.52	0.56	0.22	0.07	0.65	0.007
	Constant		-4.57	1.10				
	Variance herd level		0.49	0.42				

SE: standard error; OR: odds ratio; CI_95%_: confidence interval (95%)

The final models for tarsal lesions are presented in Table 5. For both, hair loss and ulceration, the risk for a lesion was significantly higher when the concrete stall base was covered with a rubber mat, in comparison to blank concrete. As for hair loss and ulceration at the carpus, frequent exercise outdoors significantly reduced the risk for hair loss at the tarsus. A higher feeding frequency per day decreased the risk for swelling, and a bedding depth of >2 cm had a protective effect on ulceration at the tarsus, which is similar to hair loss at the carpus. The VPC of the final tarsal models was 10.4, over 16.6, to 7.9% for the farm level for the outcomes hair loss, ulceration, and swelling, respectively.

**Table 5:** Final multilevel logistic regression model for tarsal lesions with cow and herd level risk factors in 27 tie stall farms (n = 609 cows) in Switzerland.

Tarsal lesion	Variable	Category	Coefficient	SE	OR	CI_95%_		P-value
**Hair loss**	**Month of visit**	December	Reference	-	-	-	-	-
		January	1.74	0.56	5.69	1.91	16.94	0.002
		February	2.17	0.51	8.79	3.24	23.90	<0.001
		March	0.40	0.53	1.49	0.53	4.19	0.452
		April	-0.57	0.74	0.57	0.13	2.40	0.440
	**Body Condition Score**	≤ 2.75	Reference	-	-	-	-	-
		3.0	-0.04	0.27	0.96	0.56	1.64	0.875
		3.25	-0.78	0.32	0.46	0.24	0.85	0.014
		>3.25	-1.09	0.37	0.34	0.16	0.70	0.003
	**Stall base**	concrete	Reference	-	-	-	-	-
		rubber mat	1.66	0.45	5.24	2.17	12.65	<0.001
	**Outdoor access (days/month)**	< 13	Reference	-	-	-	-	-
		13	-1.27	0.53	0.28	0.10	0.79	0.016
		14–15	-1.40	0.55	0.25	0.08	0.72	0.010
		> 15	-1.42	0.73	0.24	0.06	1.00	0.050
	Constant		-0.29	0.66				
	Variance herd level		0.38	0.21				
**Ulceration**	**Stall base**	concrete	Reference	-	-	-	-	-
		rubber mat	1.65	0.52	5.23	1.88	14.52	0.002
	**Bedding depth (point B)**	≤2 cm	Reference	-	-	-	-	-
		>2 cm	-0.66	0.32	0.52	0.28	0.96	0.037
	Constant		-1.93	0.52				
	Variance herd level		0.66	0.28				
**Swelling**	**Cow Cleanliness**	0	Reference	-	-	-	-	-
		1–2	-0.16	0.31	0.85	0.46	1.57	0.603
		3–4	0.59	0.33	1.81	0.95	3.45	0.073
		>4	0.95	0.34	2.59	1.33	5.02	0.005
	**Rear curb height**	≤13 cm	Reference	-	-	-	-	-
		13–19 cm	-0.50	0.40	0.61	0.28	1.33	0.213
		20–25 cm	-0.11	0.37	0.90	0.44	1.85	0.773
		>25 cm	-1.24	0.43	0.29	0.12	0.67	0.004
	**Feeding frequency (count/day)**	≤2	Reference	-	-	-	-	-
		≥3	-0.74	0.33	0.48	0.25	0.92	0.027
	Constant		-0.91	0.37				
	Variance herd level		0.28	0.17				

SE: standard error; OR: odds ratio; CI_95%_: confidence interval (95%)

Because of the low cow level prevalence of stifle lesions, especially of ulceration and swelling, only univariable associations between the different stifle lesion types and potential risk factors were investigated (Table 6). Overall, the presence of a rear rail had a protective effect on all types of stifle lesions, and cows with a higher parity were at a lower risk to present with hair loss or ulceration at the stifle.

**Table 6:** Univariable multilevel logistic regression models for stifle lesions with the random effect farm in 27 tie stall farms (n = 609 cows) in Switzerland.

Stifle lesion	Variable	Category	Coefficient	SE	OR	CI_95%_		P-value
**Hair loss**	**Breed**	dairy	Reference	-	-	-	-	-
		dual-purpose	-1.37	0.54	0.25	0.09	0.73	0.011
		crossbreed	-0.64	0.33	0.53	0.27	1.02	0.056
	**Parity**	1	Reference	-	-	-	-	-
		2–3	0.09	0.30	1.09	0.61	1.96	0.774
		4	0.30	0.36	1.35	0.66	2.75	0.408
		>4	-1.02	0.39	0.36	0.17	0.77	0.009
	**Stall base**	concrete	Reference	-	-	-	-	-
		rubber mat	1.95	0.81	7.06	1.43	34.74	0.016
	**Rear rail**	absent	Reference	-	-	-	-	-
		present	-1.24	0.51	0.29	0.11	0.78	0.014
	**Withers height/bed length (ratio)**	≤0.71	Reference	-	-	-	-	-
		>0.71 - ≤0.74	1.38	0.51	3.96	1.45	10.84	0.007
		>0.74 - ≤0.77	0.98	0.56	2.67	0.90	7.95	0.078
		>0.77	0.34	0.58	1.40	0.45	4.41	0.565
**Ulceration**	**Parity**	1	Reference	-	-	-	-	-
		2–3	-0.07	0.38	0.93	0.44	1.98	0.857
		4	-0.25	0.52	0.78	0.28	2.17	0.635
		>4	-1.27	0.57	0.28	0.09	0.86	0.025
	**Bedding depth (point B)**	≤2 cm	Reference	-	-	-	-	-
		>2 cm	-2.14	0.83	0.12	0.02	0.59	0.010
	**Bedding type**	other	Reference	-	-	-	-	-
		straw	-1.07	0.54	0.34	0.12	0.98	0.045
	**Rear rail**	absent	Reference	-	-	-	-	-
		present	-1.70	0.62	0.18	0.05	0.61	0.006
**Swelling**	**Body Condition Score**	≤2.75	Reference	-	-	-	-	-
		3.0	-1.40	0.62	0.25	0.07	0.83	0.024
		3.25	-2.50	1.14	0.08	0.01	0.76	0.028
		>3.25	-1.84	1.19	0.16	0.02	1.63	0.122
	**Stall wetness**	dry	Reference	-	-	-	-	-
		moist	0.31	1.28	1.36	0.11	16.88	0.810
		wet	3.41	1.24	30.41	2.67	346.93	0.006
	**Rear rail**	absent	Reference	-	-	-	-	-
		present	-2.58	1.01	0.08	0.01	0.55	0.011

SE: standard error; OR: odds ratio; CI_95%_: confidence interval (95%)

## Discussion

We found that the highest cow level prevalence among the study population occurs for tarsal skin lesions, which were more likely when the cows were housed in stalls with rubber mats as a base and less likely, when the cows had access to frequent exercise outdoors. The moderate to high cow and herd level prevalence of the investigated skin lesions indicates, that skin lesions are a common issue on Swiss tie stall farms. Anyhow, the fact that some farms enrolled in the current study had no cow with a severe skin lesion indicates that it is realistic to largely reduce skin lesion prevalence.

In our study, skin lesions at the tarsal joint were most prevalent on the cow level. This is supported by most studies that report the tarsal joint as the location the most likely to develop skin lesions in dairy cows kept in tie stalls [9, 12, 13, 27]. Nevertheless, the comparison of previously published prevalence data though is difficult, because up to today, there is no widely accepted standard scoring system for skin lesions [47], and the investigated study populations largely differ. Hair loss is of less relevance regarding animal health and welfare [31, 46]; however, it can be seen as an indicator of mild abrasion [37] and one of the first clinical signs of repetitive trauma. The current study is the first to our knowledge that applied the lesion scoring system according to Potterton et al. [29] in tied dairy cows, and provides information on early and mild clinical signs of skin lesions, as well as of lesions that directly compromise animal welfare by their severity.

The prevalence of hair loss at the tarsus is higher in the current study (62.4%) compared to a previous study that used the same scoring system (40.1%, [29]). In our study population, the prevalence of ulceration and swelling at the tarsus is lower than the minimum reported tarsal lesion prevalence in Canadian tie stall herds [9–12]. The prevalence of ulceration and swelling at the carpus on average seems to be lower in Swiss compared to Canadian tie stalls [9–12]. One reason for this difference might be that the cows enrolled in the current study had almost daily access to pasture in summer and at least some outdoor exercise in winter, which is required by the Swiss animal welfare legislation [32]. Additionally, our study population consisted of two thirds either dual-purpose or crossbreeds that are suspected to be less vulnerable to develop skin lesions compared to higher-yielding breeds [14, 26], although this association could not be statistically verified in our study.

The prevalence of stifle lesions was unknown in Switzerland, and only a few cases were reported and further investigated earlier [48]. Although the prevalence seems to be low, clinical signs with a relatively small diameter might cause severe macroscopic and microscopic damage in the underlying tissue, involving synovial bursae, tendons, and ligaments [48], and therefore interfere with animal welfare by causing inflammation and pain. These severe lesion-effects may result in early culling [48]. The moderate to high herd level prevalence of hair loss and ulceration indicates that lesions of the skin above the stifle might appear more frequently than expected.

Across all lesion types and locations, factors covering for the variety of bedding quantity, quality and the stall base often showed a significant association with the assessed outcomes. The use of rubber mats in comparison to concrete increased the odds of hair loss and ulceration at the tarsus, as well as of hair loss at the stifle, which stands in agreement with previous studies [12, 37]. The relatively hard and resilient rubber mats often have a non-sufficient cushioning-effect, and might lead to abrasion and burnings of the skin, when the cow lies down or stands up [49]. Jewell et al. [11] and Lim et al. [47] reported how farmers seem to expect an insufficient cushioning-effect when the stall base is concrete, and use a higher bedding quantity to cover it, compared to rubber mats. In our study, we found no correlation between stall base and bedding quantity, thus we assume the increased risk to originate from the rubber mats themselves, and not to be confounded by a lower bedding quantity used. The provision of >2 cm bedding depth had a significant protective effect on carpal hair loss, tarsal and stifle ulceration, which is consistent to previous results of other research groups [14, 50]. Especially in tie stalls, where cows have limited choice where and how to lie down, an adequate amount of bedding should be provided at any time, which probably requires the supply of fresh bedding material multiple times each day.

According to our results, we recommend farmers the replacement of the present rubber mats with whole straw deep bedding to further reduce the skin lesion prevalence in their tied dairy herd. Whole straw is most commonly used in Swiss tie stalls, and has a positive effect on skin integrity [25, 31, 37]. An alternative would be the use of sand as bedding material that was shown to be associated with a lower odds for skin lesions [4, 22], but its use is uncommon in Switzerland and hence, the availability of a product of adequate quality and its recycling might be challenging.

Among the tested management associated risk factors, either a more frequent exercise outdoors or a more frequent feed supply reduced the odds for hair loss and ulceration at the carpus, as well as of hair loss and swelling at the tarsus. Both factors represent an opportunity for the cow to change posture. After the cow changes its posture from lying to standing, the punctual pressure on a particular skin region [49] is dissolving, so that the blood supply to the skin and its surrounding tissue can return to normal. This prevents the skin from microscopic and macroscopic ischemic necrosis and supports the healing of existing lesions. The positive effect of frequent exercise outdoors stands in agreement to the findings of previous research [1, 14, 19]. The outdoor yards the cows have access to throughout winter are mostly fitted with slatted floors or whole concrete or gravel, all of them representing surfaces of a somewhat abrasive and relatively hard quality. Still, the positive effect of the exercise outdoors seems to be predominant, as the cows are usually standing or walking but not lying in the outdoor pens. However, a potential negative influence of a poor yard quality on other animal welfare aspects, e.g. claw health, is out of the scope of the present study and should be addressed in the future.

All types of stifle lesions were negatively associated with the presence of a rear rail, indicating that its purpose in the respective stalls is met. A rear rail is a stall design feature of traditional Swiss tie stalls that is installed to motivate the cow to lie down further forward in the stall and to keep the bedding at its place. A statistical association between bedding depth and rear rail presence was not detected in the current study, although it was expected. Therefore, the main effect of the rear rail seems to be in the positioning of the cows when they are lying down. Lying down on top of the rear rail might be uncomfortable or painful for the cow. The contact of the cows’ legs to a rear rail while lying can be suspected to cause abrasion and swelling at the tarsal joint, which has already been shown for contact to the rear curb [23, 51]. When the cows are lying down further forward in the stall, the probability that the lying surface in this area is covered with a sufficient amount of dry and clean bedding is increased so that skin lesions can be prevented.

The reported effects of the cow cleanliness on skin lesions are not entirely consistent throughout literature. Some studies reported a protective effect on lesions, when the cows were dirtier [11, 29], yet the contrary effect was shown for carpal [11] and tarsal lesions [30, 47]. In the current study, dirtier cows were at a higher risk for swellings at the tarsus. The persistent exposure of the skin to excrements irritates the skin [52] and may lead to inflammation and concomitant swelling, especially when an open wound or ulceration is present. Still, the magnitude of the influence shown by the model was unexpected, as the cut-off of the category serving for the dirtiest twenty-five percent of cows in the study population was still relatively low. This leads to the suggestion, that even a slight coverage of certain skin areas with manure and urine marks a significant hygiene issue and the cleanliness of cows and lying surfaces in tie stalls should be focused in preventing skin lesion development.

In the current study, the group of cows with a parity >4 was at the lowest risk to present hair loss and ulceration at the stifle, while no association was represented in the final models for carpal and tarsal lesions. In contrast to our findings, an increased parity or age of the cow is consistently reported to increase the risk for skin lesions of several types and locations [14, 20, 51]. One reason that the results of the current study differ from those findings might be that pregnant heifers in Switzerland are traditionally managed in pasture-based systems and are housed for the first time a few weeks prior to first calving. These heifers need to adapt to the tie stall environment for the first time and might therefore be more likely to develop skin lesions compared to older cows that are already habituated.

In contrast to many other studies, lactating and dry cows were included in the sampling process of the current study; nevertheless, in the final multilevel models of the current study that were selected for their biological relevance, the lactation status was not associated with skin lesions. This might be explained by the fact that lactating and dry cows in traditional Swiss tie stalls are mainly kept under the same housing conditions, which were expected to account for a majority of the variation in skin lesion prevalence. Still, the proportion of dry cows in the study population was low, so that a potential influence cannot be completely rejected and more research would be necessary to exclude potential associations.

Lameness was not associated with any skin lesion type in the current study, although, it is one of the most frequently reported risk factors associated with skin lesions at cows' legs [20, 25]. The prevalence of lameness among the study population was low regarding both applied lameness scoring systems. Especially the more detailed locomotion scoring [43] mainly revealed mild lameness and rarely clinical or severe lameness. Therefore, the behavioral impairment of mildly lame cows may not be strong enough to result in a significant association with skin lesions. However, if the lameness prevalence or the mean locomotion score across herds were higher, a detectable association between lameness and skin lesions would be expected. Furthermore, claw lesions were not evaluated in the current study, thus a potential confounding effect of claw health on the association of lameness and skin lesions cannot be fully excluded. Nevertheless, claw lesions are the cause of about 90% of lameness cases [53], so that lameness should serve as an adequate dummy for distal limb pathologies.

All farmers participated voluntarily, so a potential selection bias cannot be fully excluded, which might have led to an underestimation of the cow and herd level prevalence of skin lesions, because severely compromised farms would possibly not participate. In spite of being a convenience sample, the distribution of the studies' farms among the cantons reflects the main locations of traditional Swiss tie stalls. Besides dairy breeds, crossbreeds and dual-purpose breeds were included in the current study, because the housing of these breeds is characteristic for Switzerland. The mean herd size was 26 cows (median herd size of 22 cows) in the investigated herds and is thus slightly higher but very close to the average Swiss dairy herd, including both free and tie stalls, where a mean herd size of 22 cows per herd [54] is reported. For each skin lesion type and location, the CI_95%_ of the cow level prevalence was narrow, which indicates a good reliability of the current results and an adequate sample size on the cow level, ensuring comparability and data quality. Regarding the rather broad CI_95%_ of the herd level prevalence of the outcomes, further investigations enrolling a larger number of farms might be suggested to ensure a more precise prevalence estimation. Still, as the aim of this study primarily focused on measurements on the cow level, the rather small number of enrolled farms is expected to be of minor importance, especially as in the final multilevel models a high power was reached.

## Conclusions

Skin lesions at the carpus, tarsus, and stifle are a common issue in dairy cows kept in traditional Swiss tie stalls. Although dairy facilities among Switzerland are continuously adapted according to national guidelines and recommendations of animal welfare labels, the prevalence of tarsal lesions seems to have slightly increased throughout the last decade. In all enrolled farms, at least mild signs of abrasion at the tarsus of the examined cows were recorded and the vast majority of farms housed at least one cow with a lesion type and severity that is suspected to compromise animal welfare. Most of the risk factors that were shown to be associated with skin lesions have the potential to be improved by slight, but effective management changes, for instance by the provision of frequent exercise outdoors or the use of more bedding material. The more days per month the cows had the possibility to exercise outdoors, the smaller the odds of developing several lesion types got. Regarding tie stall design, cows that were kept on solid rubber mats and a small amount of bedding material were at a higher risk to develop skin lesions than those housed on a well-cushioned lying surface. Especially during the winter season, a high quality of the lying surface and stall design features is required, as the cows spend most of the time indoors. The longitudinal effect of improving the distinguished risk factors should be addressed in future studies with an experimental study design and a longitudinal follow-up of individual cows.

## Supporting information

S1 Fig Example of one laminated definition card. (PDF)

S2 Fig Selected stalls for quantitative measurements. (PDF)

S3 Fig Schematic illustration of stall measurements. (PDF)

S1 Table Non-selected regression models. (DOCX)

S2 Table Correlation matrix. (XLSX)

S1 Dataset Animal and risk factor related Excel data. (XLSX)
