# Supplementary material for: Carpal, tarsal, and stifle skin lesion prevalence and potential risk factors in Swiss dairy cows kept in tie stalls: A cross-sectional study
Source: PLoS One. 2020 Feb 12;15(2):e0228808. doi: 10.1371/journal.pone.0228808 (PMC7015392; doi:10.1371/journal.pone.0228808)
Supplement: S1 Fig — (PDF) [file pone.0228808.s001.pdf]

**Tarsus**	**Hair loss** (HL)	**Ulceration** (UL)	**Swelling**(SW)
**Score 0**	no lesion 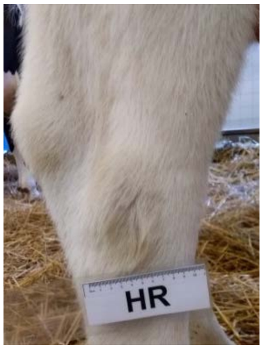	no lesion 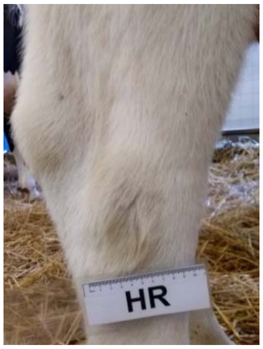	no swelling 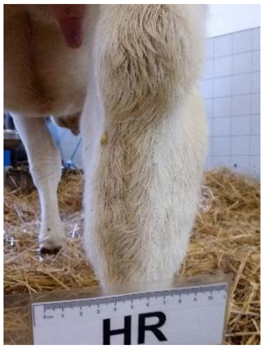
**Score 1**	ø < 2 cm 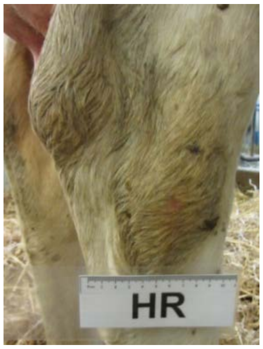	ø < 2 cm 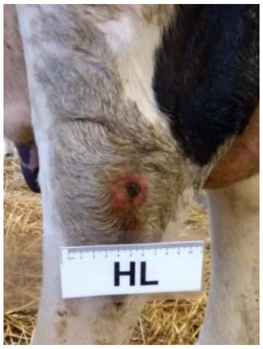	thicker than normal 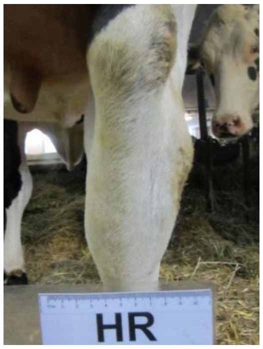
**Score 2**	ø 2 - 2.5 cm 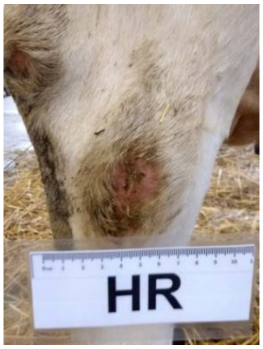	ø 2 - 2.5 cm 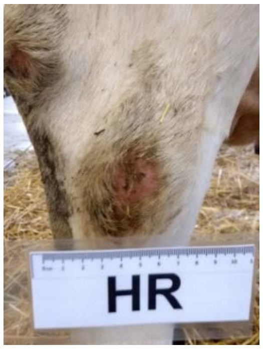	obvious swelling 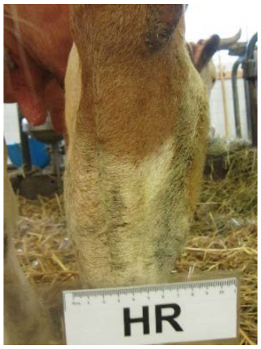
**Score 3**	ø > 2.5 cm 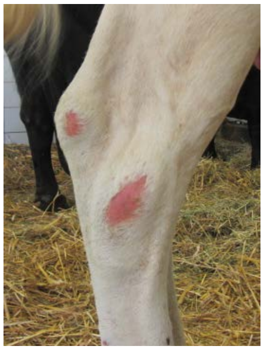	ø > 2.5 cm 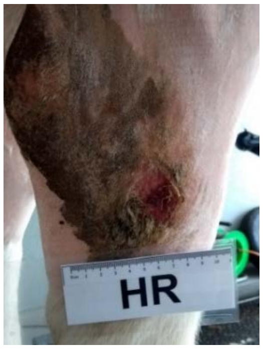	extensive swelling 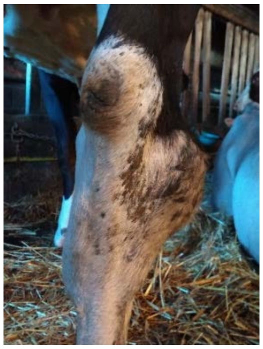

S1 Fig. Example of one laminated definition card. Tarsal lesion scoring.
