# Supplementary material for: Carpal, tarsal, and stifle skin lesion prevalence and potential risk factors in Swiss dairy cows kept in tie stalls: A cross-sectional study
Source: PLoS One. 2020 Feb 12;15(2):e0228808. doi: 10.1371/journal.pone.0228808 (PMC7015392; doi:10.1371/journal.pone.0228808)
Supplement: S2 Fig — (PDF) [file pone.0228808.s002.pdf]

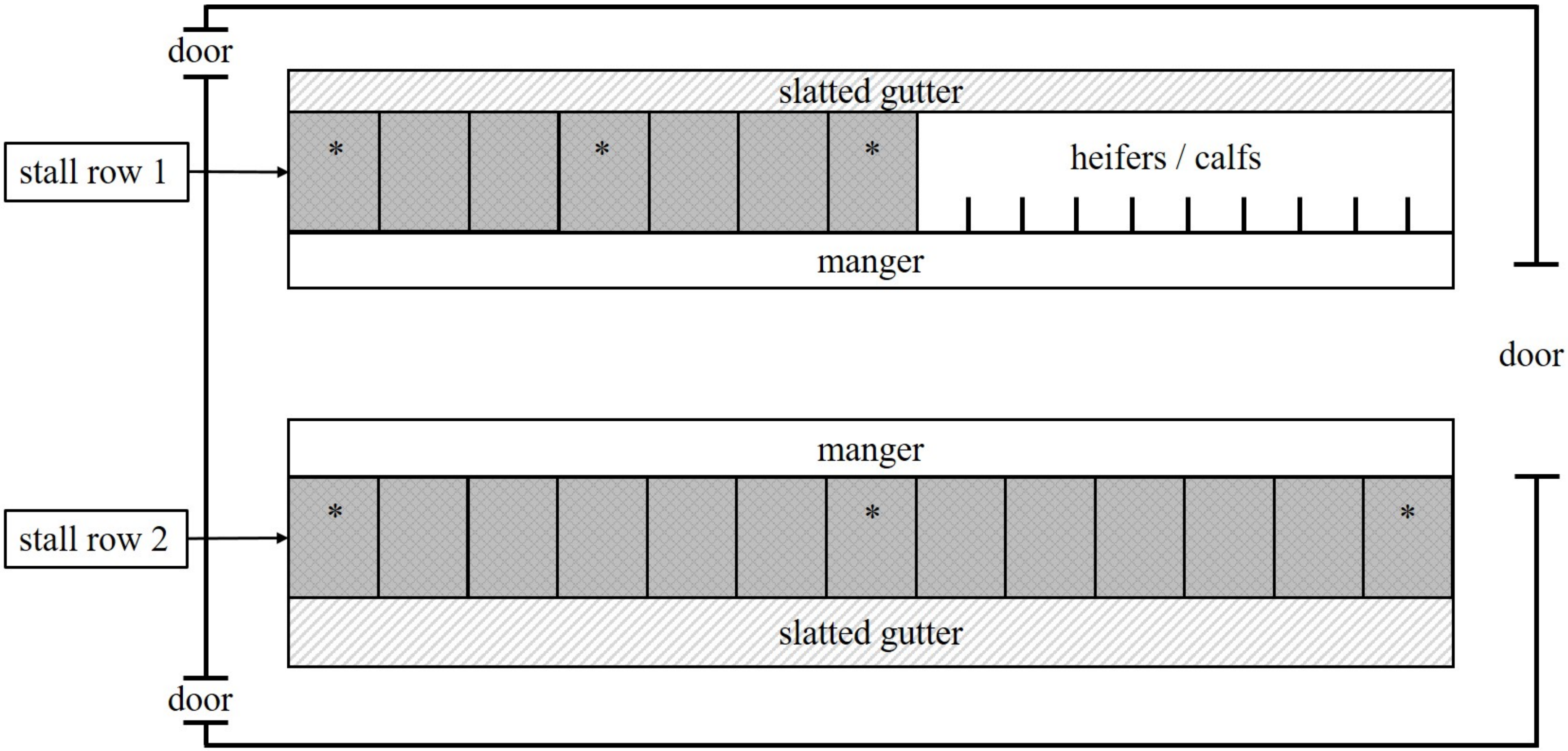


**S2 Fig. Selected stalls for quantitative measurements.** Schematic illustration of a top view of a typical Swiss tie stall with two different stall rows. The asterisk (*) indicates stalls selected for quantitative measurements.
