# Supplementary material for: Carpal, tarsal, and stifle skin lesion prevalence and potential risk factors in Swiss dairy cows kept in tie stalls: A cross-sectional study
Source: PLoS One. 2020 Feb 12;15(2):e0228808. doi: 10.1371/journal.pone.0228808 (PMC7015392; doi:10.1371/journal.pone.0228808)
Supplement: S3 Fig — (PDF) [file pone.0228808.s003.pdf]

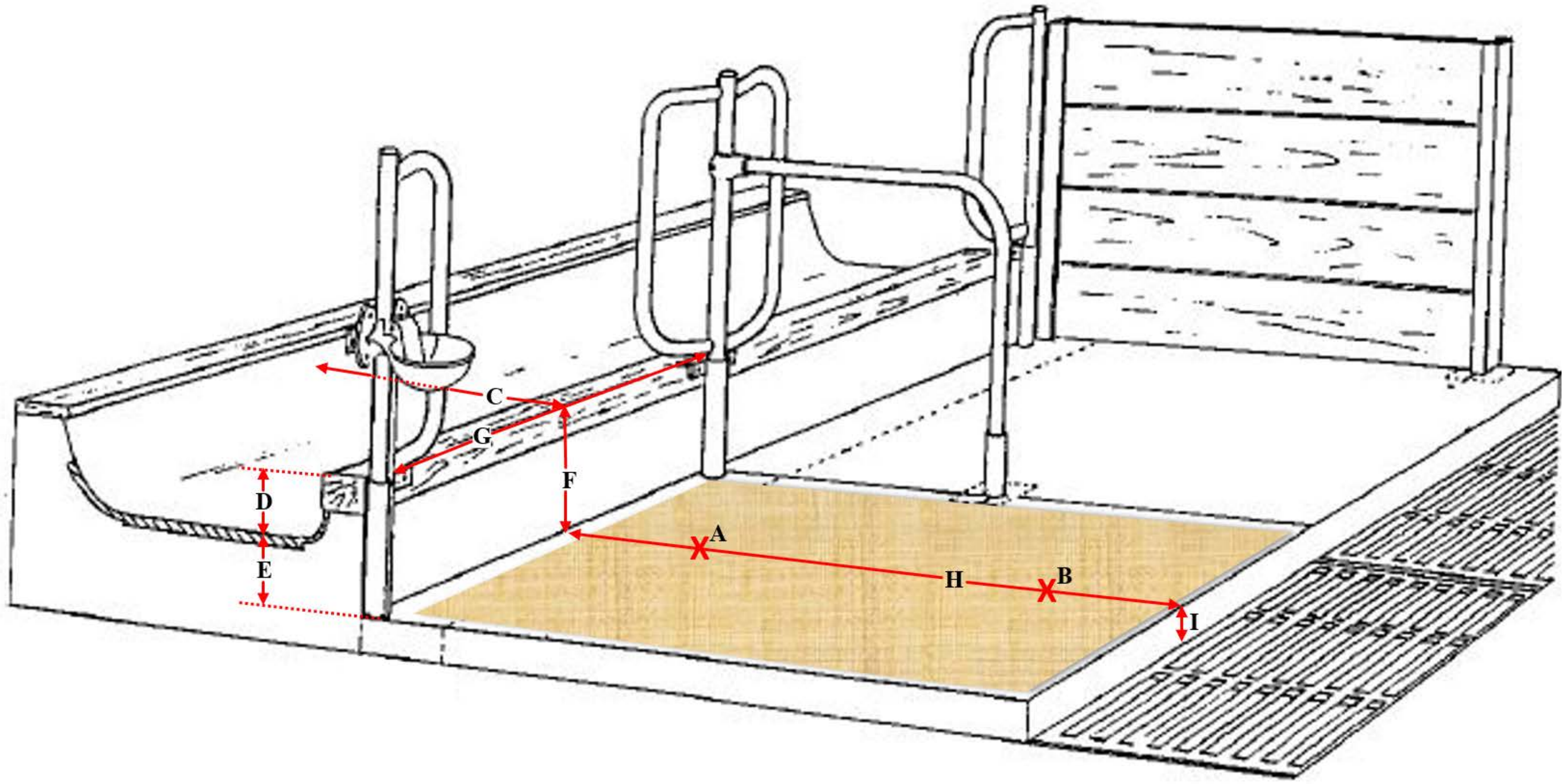

S3 Fig. Schematic illustration of stall measurements. Measurements taken in selected stalls.
(A) point A: assessment of bedding depth and stall wetness; (B) point B: assessment of bedding depth and stall wetness; (C) free lunge space; (D) manger depth; (E) manger base height; (F) manger wall height; (G) bed width; (H) bed length; (I) rear curb height.

Illustration adapted from Swiss Federal Food Safety and Veterinary Office. Fachinformation Tierschutz, Vorschläge für einfache Anpassungen in Anbindeställen für Kühe. 2013.
