# Supplementary material for: Carpal, tarsal, and stifle skin lesion prevalence and potential risk factors in Swiss dairy cows kept in tie stalls: A cross-sectional study
Source: PLoS One. 2020 Feb 12;15(2):e0228808. doi: 10.1371/journal.pone.0228808 (PMC7015392; doi:10.1371/journal.pone.0228808)
Supplement: S1 Table — (DOCX) [file pone.0228808.s004.docx]

**S4 Table. Non-selected regression models.** Statistically valid logistic regression models that dropped out during the model selection process.

| **Outcome** | **Variable** | **Category** | **Coefficient** | **SE** | **OR** | **CI_95%_** | | **P-value** |
| --- | --- | --- | --- | --- | --- | --- | --- | --- |
| **Hair loss (Carpus)** | **Month of visit** | December | Reference | - | - | - | - | - |
|  |  | January | 2.03 | 0.52 | 7.58 | 2.75 | 20.90 | <0.001 |
|  |  | February | 1.79 | 0.47 | 6.01 | 2.40 | 15.03 | <0.001 |
|  |  | March | 1.51 | 0.50 | 4.54 | 1.72 | 12.02 | 0.002 |
|  |  | April | 1.50 | 0.61 | 4.48 | 1.36 | 14.75 | 0.014 |
|  | **Bedding depth (point A)** | ≤2 cm | Reference | - | - | - | - | - |
|  |  | >2 cm | -1.01 | 0.38 | 0.36 | 0.17 | 0.76 | 0.007 |
|  | **Manger wall depth** | ≤15.5 cm | Reference | - | - | - | - | - |
|  |  | 15.6 – 17.5 cm | 0.20 | 0.31 | 1.22 | 0.66 | 2.25 | 0.524 |
|  |  | 17.6 – 20 cm | 0.30 | 0.32 | 1.35 | 0.71 | 2.53 | 0.358 |
|  |  | >20 cm | -1.20 | 0.45 | 0.30 | 0.13 | 0.73 | 0.008 |
|  | Constant |  | -1.02 | 0.47 |  |  |  |  |
|  | Variance herd level |  | 0.28 | 0.15 |  |  |  |  |
| **Ulceration (Carpus)** | **Month of visit** | December | Reference | - | - | - | - | - |
|  |  | January | 3.22 | 1.41 | 25.01 | 1.57 | 397.84 | 0.023 |
|  |  | February | 2.19 | 1.30 | 8.96 | 0.70 | 115.14 | 0.092 |
|  |  | March | 3.66 | 1.28 | 38.84 | 3.14 | 481.06 | 0.004 |
|  |  | April | 3.05 | 1.41 | 21.18 | 1.35 | 333.21 | 0.030 |
|  | **Free lunge space** | ≤73 cm | Reference | - | - | - | - | - |
|  |  | >73 cm | -1.71 | 0.58 | 0.18 | 0.06 | 0.57 | 0.004 |
|  | **Lactation stage** | dry cow | Reference | - | - | - | - | - |
|  |  | lactating cow | -1.14 | 0.52 | 0.32 | 0.12 | 0.88 | 0.028 |
|  | **Label** | no | Reference | - | - | - | - | - |
|  |  | yes | -1.28 | 0.59 | 0.28 | 0.09 | 0.88 | 0.030 |
|  | Constant |  | -3.36 | 1.25 |  |  |  |  |
|  | Variance herd level |  | 0.79 | 0.52 |  |  |  |  |

| **Swelling (Carpus)** | **Label** | no | Reference | - | - | - | - | - |
| --- | --- | --- | --- | --- | --- | --- | --- | --- |
|  |  | yes | -1.11 | 0.52 | 0.33 | 0.12 | 0.91 | 0.033 |
|  | **Free lunge space** | ≤73 cm | Reference | - | - | - | - | - |
|  |  | >73 cm | -1.54 | 0.55 | 0.21 | 0.07 | 0.63 | 0.005 |
|  | Constant |  | -2.05 | 0.41 |  |  |  |  |
|  | Variance herd level |  | 0.55 | 0.50 |  |  |  |  |
| **Hair loss (Tarsus)** | **Month of visit** | December | Reference | - | - | - | - | - |
|  |  | January | 2.15 | 0.64 | 8.60 | 2.44 | 30.36 | 0.001 |
|  |  | February | 3.05 | 0.65 | 21.07 | 5.84 | 76.03 | <0.001 |
|  |  | March | 1.42 | 0.60 | 4.14 | 1.27 | 13.50 | 0.018 |
|  |  | April | 0.50 | 0.71 | 1.65 | 0.41 | 6.59 | 0.477 |
|  | **Stall base** | concrete | Reference | - | - | - | - | - |
|  |  | rubber mat | 1.17 | 0.46 | 3.21 | 1.29 | 7.98 | 0.012 |
|  | **Body Condition Score** | ≤2.75 | Reference | - | - | - | - | - |
|  |  | 3.0 | -0.01 | 0.28 | 0.99 | 0.57 | 1.70 | 0.966 |
|  |  | 3.25 | -0.82 | 0.33 | 0.44 | 0.23 | 0.83 | 0.012 |
|  |  | >3.25 | -0.99 | 0.37 | 0.37 | 0.18 | 0.77 | 0.007 |
|  | **Rear step height** | ≤13 cm | - | - | - | - | - | - |
|  |  | 13 – 19.25 cm | -0.97 | 0.55 | 0.38 | 0.13 | 1.11 | 0.077 |
|  |  | 19.26 – 25.0 cm | -0.78 | 0.49 | 0.46 | 0.18 | 1.20 | 0.111 |
|  |  | >25.1 cm | -1.02 | 0.48 | 0.36 | 0.14 | 0.93 | 0.034 |
|  | Constant |  | -0.94 | 0.64 |  |  |  |  |
|  | Variance herd level |  | 0.59 | 0.31 |  |  |  |  |
| **Ulceration (Tarsus)** | **Month of visit** | December | Reference | - | - | - | - | - |
|  |  | January | 0.03 | 0.71 | 1.03 | 0.26 | 4.18 | 0.963 |
|  |  | February | 1.22 | 0.61 | 3.37 | 1.02 | 11.21 | 0.047 |
|  |  | March | 1.38 | 0.63 | 3.97 | 1.15 | 13.76 | 0.030 |
|  |  | April | 0.75 | 0.77 | 2.13 | 0.47 | 9.66 | 0.329 |
|  | **Stall base** | concrete | Reference | - | - | - | - | - |
|  |  | rubber mat | 1.35 | 0.55 | 3.85 | 1.30 | 11.40 | 0.015 |
|  | **Rear rail** | absent | Reference | - | - | - | - | - |
|  |  | present | -0.88 | 0.39 | 0.42 | 0.20 | 0.89 | 0.024 |
|  | Constant |  | -2.12 | 0.71 |  |  |  |  |
|  | Variance herd level |  | 0.75 | 0.33 |  |  |  |  |
| **Ulceration (Tarsus)** | **Stall base** | concrete | Reference | - | - | - | - | - |
|  |  | rubber mat | 1.78 | 0.49 | 5.93 | 2.27 | 15.52 | <0.001 |
|  | **Bedding depth (point B)** | ≤2 cm | Reference | - | - | - | - | - |
|  |  | >2 cm | -0.73 | 0.31 | 0.48 | 0.26 | 0.89 | 0.019 |
|  | **Outdoor access (days/month)** | <13 | Reference | - | - | - | - | - |
|  |  | 13 | -0.47 | 0.42 | 0.62 | 0.28 | 1.41 | 0.257 |
|  |  | 14 - 15 | -0.71 | 0.52 | 0.49 | 0.18 | 1.36 | 0.171 |
|  |  | >15 | -1.35 | 0.59 | 0.26 | 0.08 | 0.82 | 0.021 |
|  | Constant |  | -1.50 | 0.55 |  |  |  |  |
|  | Variance herd level |  | 0.47 | 0.23 |  |  |  |  |
| **Swelling (Tarsus)** | **Month of visit** | December | Reference | - | - | - | - | - |
|  |  | January | -0.45 | 0.41 | 0.64 | 0.29 | 1.43 | 0.279 |
|  |  | February | 0.17 | 0.37 | 1.18 | 0.58 | 2.42 | 0.649 |
|  |  | March | -0.88 | 0.40 | 0.42 | 0.19 | 0.90 | 0.027 |
|  |  | April | -0.53 | 0.53 | 0.59 | 0.21 | 1.67 | 0.320 |
|  | **Rear rail** | absent | Reference | - | - | - | - | - |
|  |  | present | -0.78 | 0.26 | 0.46 | 0.28 | 0.76 | 0.002 |
|  | **Slatted gutter** | absent | Reference | - | - | - | - | - |
|  |  | present | 0.67 | 0.28 | 1.96 | 1.13 | 3.40 | 0.017 |
|  | **Cow Cleanliness Score** | 0 | Reference | - | - | - | - | - |
|  |  | 1 – 2 | -0.12 | 0.31 | 0.88 | 0.49 | 1.61 | 0.686 |
|  |  | 3 – 4 | 0.71 | 0.32 | 2.04 | 1.10 | 3.78 | 0.024 |
|  |  | >4 | 1.20 | 0.32 | 3.33 | 1.79 | 6.19 | <0.001 |
|  | Constant | -1.35 | 0.41 |  |  |  |  |  |
|  | Variance herd level | 0.11 | 0.13 |  |  |  |  |  |

SE: standard error; OR: odds ratio; CI_95%_: confidence interval (95%)
